# ACHIEVING UNIVERSAL HEALTH COVERAGE FOR OLDER ADULTS: A REVIEW OF GHANA’S NATIONAL HEALTH INSURANCE SCHEME

**DOI:** 10.1093/geroni/igac059.2144

**Published:** 2022-12-20

**Authors:** Samuel Asante, Grace Karikari

**Affiliations:** Northeastern State University, Broken Arrow, Oklahoma, United States; University of North Dakota, Grand Forks, North Dakota, United States

## Abstract

Universal Health Coverage (UHC) is defined by the World Health Organization (WHO) as ensuring that all people receive quality health services they need without financial hardship. Predicated on the idea of providing universal healthcare to all Ghanaians, Ghana’s National Health Insurance Scheme (NHIS), established in 2003, has done considerably well among some age categories than others. While most studies on this healthcare program have focused on the health-seeking behaviors among users, its implementation, financial sustainability, and the role of national politics in its administration, little has been done to understand the program’s effectiveness in achieving a universal health coverage for and its impact on older Ghanaians. Consistent with WHO’s objective of achieving UHC, as enshrined in the Global Strategy on Aging and Health, this review examines Ghana’s progress toward achieving UHC for all. The paper specifically provides a narrative review of the National Health Insurance Scheme in advancing the interest and welfare of older Ghanaians. Findings revealed that not all individuals considered as older adults, either conventionally or legally, are beneficiaries of the programs; that WHO’s objective of obtaining needed care without financial hardship may be far from reach for most older Ghanaians; and the program only offers basic protection to current beneficiaries. We address these areas of concern to achieving UHC. We also make recommendations for a path forward where all stakeholders—older adults, families, healthcare providers, and policymakers—involved can play a role in ensuring all eligible older adults get the quality of health care they deserve.

